# Leveraging public AI tools to explore systems biology resources in mathematical modeling

**DOI:** 10.1038/s41540-025-00496-z

**Published:** 2025-02-04

**Authors:** Meera Kannan, Gabrielle Bridgewater, Ming Zhang, Michael L. Blinov

**Affiliations:** 1 Center for Cell Analysis and Modeling, UConn Health, Farmington, CT 06030 USA; 2 Theoretical Biology and Biophysics Group, Los Alamos National Laboratory, Los Alamos, NM 87544 USA

**Keywords:** Systems biology, Computer modelling, Dynamic networks, Dynamical systems, Information theory, Software, Standardization, Systems analysis

## Abstract

Predictive mathematical modeling is an essential part of systems biology and is interconnected with information management. Systems biology information is often stored in specialized formats to facilitate data storage and analysis. These formats are not designed for easy human readability and thus require specialized software to visualize and interpret results. Therefore, comprehending modeling and underlying networks and pathways is contingent on mastering systems biology tools, which is particularly challenging for users with no or little background in data science or system biology. To address this challenge, we investigated the usage of public Artificial Intelligence (AI) tools in exploring systems biology resources in mathematical modeling. We tested public AI’s understanding of mathematics in models, related systems biology data, and the complexity of model structures. Our approach can enhance the accessibility of systems biology for non-system biologists and help them understand systems biology without a deep learning curve.

## Introduction

Biotechnologies, such as next-generation sequencing, high-throughput screening, single-cell RNA sequencing, and mass cytometry, have generated a vast amount of biological and biomedical data. Systems biology integrates these data from various sources through analysis and modeling from multiple cross-linked databases and software tools. Usually, the data are archived in formats that are designed for data storage and analysis purposes, such as a variety of XML formats for describing different types of mathematical models, such as Systems Biology Markup Language (SBML)^1^ and related constructs such as pathways coded in Biological PAthway eXchange format (BioPAX)^2^ and visual diagrams coded in Systems Biology Graphical Notations format (SBGN)^3^. These formats, however, are not designed for human readability and, therefore, necessitate specialized software for proper visualization and result interpretation^4–11^. Meanwhile, comprehension and proper use of systems biology resources require extensive data-processing technologies, making system biology a difficult subject for biologists and/or students who do not have a data science background. To these users, programming, data formats, and mathematical equations and models belong to a different realm of expertise. Here, we introduce an approach leveraging public AI tools (ChatGPT, Perplexity, MetaAI, etc.) to explore various aspects of systems biology, specifically mathematical models. We also discuss the advantages and disadvantages of our approach.

## Results

### Mathematical modeling of system biology

Mathematical modeling is crucial in systems biology, which studies how the components of biological systems interact with each other. Mathematical models are widely adopted across disciplines, from pharmacology and pharmacokinetics^12^ to personalized models for cancer^13^, highlighting their cross-cutting importance in scientific research. For example, it has been shown to bridge the gap between existing clinical knowledge and a mechanistic understanding of pathological processes^14^. The models are not limited to biology but have been extended to fields like chemistry and biotechnology^15^.

In this study, we consider systems biology data stored in community standards and formats supported by the “COmputational Modeling in BIology NEtwork” (COMBINE)—an initiative to coordinate the development of the various community standards and formats for computational models^16^. These standards are supported by a majority of system biology tools designed to visualize, simulate, and analyze mathematical models. For example, the Virtual Cell (VCell)^5,6^, COPASI^11^, BioNetGen^17,18^, and NEURON^19^ create and simulate mathematical models. Also, multiple databases are available to store systems biology data and mathematical models, such as BioModel^20^ and CellML^21^ databases of mathematical models, and Reactome^22^ and KEGG^23^ databases of pathways.

Despite the availability of system biology resources, understanding system biology is still challenging with a steep learning curve: The terminology, programming languages associated with different programs and interfaces, mathematical style definitions, etc. are complex and vary across different system biology tools. Furthermore, a high mathematics level is required to explore system biological modeling to its full extent. For example, differential equations, which are key in modeling biological processes^24^, require strong calculus knowledge. As a result, education in system biology is usually offered post-undergraduate. Meanwhile, biologists who do not have a data science background very often find system biology difficult, while they are keen to adopt system biology in research. To address the challenges, here we introduce an AI-aided approach to help explore system biology and modeling data with no difficult learning process.

### Data formats used in systems biology

Various data formats are used in systems biology to store data. Thanks to the “COmputational Modeling in BIology NEtwork” (COMBINE) initiative, which coordinates the development of community standards and formats for all aspects of modeling in biology, the number of data formats is relatively small compared to hundreds of system biology tools and databases. For example, the SBML format^1^, developed to exchange data in a form suitable for mathematical modeling, is supported in more than 100 modeling and simulation tools, including widely used tools like VCell and COPASI. The Biological Pathway Exchange (BioPAX) format was developed for network analysis, gene enrichment analysis, and validation and is used in multiple tools such as PaxTools^9^. The Systems Biology Graphical Notations (SBGN) format has been used for visualization and disease maps^7,25,26^. The BioNetGen Language (BNGL) is a text-based language^27^ for specifying rule-based models designed in BioNetGen software. NeuroML format^28^ is used to define and exchange descriptions of neuronal cell and network models. The CellML format^29^ is used to work with the Physiome software^30^, while Virtual Cell Markup Language (VCML) describes models done in VCell. The multitude of system biology data formats enriches data availability across various software and tools, however, it deepens the learning curve for non-systems biology, non-data science users.

### Overview of public AI tools

AI tools can be applied in system biology learning and exploration. However, most public AI tools limit free usage (Table 1), e.g. through truncating content, limiting file attachments, or rejecting to process large pieces of text. For example, Perplexity, SciSpace, and HyperWrite employ daily token systems to regulate the number of responses they can generate per day. Despite these constraints, there are ways to maximize the utility of free versions, for example by breaking larger files into smaller chunks.

**Table 1:** Summary of free AI tools that can be used in systems biology learning and exploration. Current as of July 2024

Name	Link	Free version	No sign-up	No daily limit	No limit on text	Providing references
ChatGPT	https://chatgpt.com/	•	•	•		
Meta AI	https://www.meta.ai/	•	•	•		
Gemini	https://gemini.google.com/	•		•		
HyperWrite	https://www.hyperwriteai.com/	•				
Phind	https://www.phind.com/	•		•		•
Perplexity	https://www.perplexity.ai/	•				•
You.com	https://you.com/	•		•		•
HuggingChat	https://huggingface.co/chat/	•		•		•
SciSpace	https://typeset.io/	•				•
Microsoft Copilot	https://copilot.microsoft.com/	•		•		•

Additionally, public AI tools’ requirement to register upfront can limit users’ experience due to privacy concerns. In this regard, ChatGPT and MetaAI may be appealing to use as both tools allow an infinite number of queries in a completely anonymous mode. Also, some AI tools have employed multiple ways to uncover user’s privacy. Platforms, such as Gemini, automatically create accounts for users logged into associated services like Google Chrome. Perplexity, Phind, Microsoft Copilot, and HuggingChat allow anonymous use for a few questions, after which a user is prompted to register to unlock the features.

An important feature of AI tools is providing references for generated answers, which is crucial for verifying and reproducing answers independently (Table 1). However, the accuracy and consistency of provided references vary across the public AI tools. Often, the references have little or no relevance to an inquiry question.

In addition to tools for general purposes, there are specialized ones that cater to specific domains. For example, the Deep Origin specializes in computational biology and drug discovery, but its immediate registration requirements can discourage a beginner’s use. Stability AI is a text-to-image generator. Jasper AI focuses on marketing-specific content. And Koala Chat is an AI assistant and offers consolidated answers by providing access to other AIs including ChatGPT and Claude. The diversity of AI tools and platforms presents both opportunities and challenges in educational integration.

Taken together, learning system biology is challenging. Therefore, here we introduce an easy-to-use approach for using existing public AI tools to help explore various aspects of system biology for non-system biology and non-data science learners. Understanding the capabilities and constraints of various AI platforms can certainly optimize learning and usage experiences. For example, AI typically generates slightly different responses to the same question. The variations of responses, even incorrect ones, can inspire critical thinking at the user’s end.

### Use of AI to explore data formats in mathematical modeling

Most existing AI tools are capable of recognizing different biological formats, and often can quickly provide sufficient descriptions about formats for further exploration. System biology databases and tools use different formats to present the data. For example, the Reactome database presents SBML, SBGN, BioPAX2, and BioPAX3 downloading formats. A researcher may be lost in which format to choose. AI tools, such as ChatGPT, can easily recognize some formats and facilitate understanding of the formats and their usage. By analyzing a BioPAX snippet (Supplementary Note 2), ChatGPT responded with a human-readable description of the data and a summary of the format, *“This RDF/XML snippet captures structured information about the “EGFR dimerization” pathway from Reactome in the BioPAX format, emphasizing the entities involved, their relationships, and associated metadata. It’s structured to facilitate interoperability and integration of biological pathway data across different platforms and databases.”*

We tested multiple non-human readable data formats on public AI tools. An XML-based NeuroML format is designed to describe and analyze neural system models. With the provided NeuroML files, most AI tools could analyze and interpret the format. For example, we input the NeuroML of a model that describes a neuron’s simple morphology (Supplementary Note 3). Phind responds with a description of *a simplified model of a neuron’s morphology using NeuroML 2, a standardized format for defining neural network architectures and cellular components. This model focuses on the physical structure of the neuron, particularly its soma (cell body), dendrites, and a spine, which are key components of neuronal architecture relevant to understanding neural signal propagation and synaptic transmission.”* This succinct summary would be helpful for a non-data science user to understand the given NeuroML model without deciphering the complex, non-human readable NeuroML code.

Further, we used data formats in biological networks and processes to test AI tools’ comprehension of complex formats. For example, when a model in the Systems Biology Graphical Notation (SBGN) format was provided (Supplementary Note 4), all nine AI tools’ responses varied, from not recognizing the format to varying degrees of detail about the structure of the data. Some tools, like Perplexity and Phind correctly identified and described key elements in the file, including the compartment, complexes, reactions, and processes, and concluded their analysis with the significance of the formation of EGFR dimers. This concise synopsis was detailed enough for a basic understanding of the given model, thus prompting the usage of this tool for non-system biologists to quickly gain a general knowledge of a model without prior knowledge of the SBGN format.

The BioNetGen Language (BNGL) is one of the most concise modeling formats. It usually has no annotations; all biological information is contained in abbreviated species names. With a provided BNGL file (Supplementary Note 5), all nine AI tools were able to process it. Given the limited details in the BNGL file, some AI tools made incorrect assumptions and began to stray off-topic, for example, Phind AI responded, *“feedback loop and SOS protein interactions*”, which do not exist in the given BNGL model. However, ChatGPT, Perplexity, MetaAI, and HyperWrite responded with details that correctly identified the various species and how they interacted in the models. For example, when prompted to explain the biology, ChatGPT responded with a description of molecules and their interacting sites used in the rule-based description. The response was in-depth while remaining concise, and the summary included describing the molecule types, reaction rules, seed species, and other important factors for a comprehensive answer.

Taken together, various system biology data formats, regardless simple or complex ones, are recognized by public AI tools. All tools can provide human-readable descriptions of the formats, but cross-validation is recommended to ensure accurate responses.

### Use of AI to explore mathematics in system biology models

System biology modeling papers assume some mathematical background for readers, thus papers often do not explain mathematical formulas and/or reasons for selecting certain mathematical expressions. Resultingly, a novice in modeling can easily be confused by math expressions and selection of mathematical formulas in describing changes in biological parameters and effects (stimulation or inhibition) modeled by this mathematics. To alleviate these difficulties, we tested the extent to which AI can assist in understanding the mathematical expressions used in system biology.

As one example, we used a recent paper on Aging Phenotypes^31^. This paper describes the timeline of human aging as a function of measured amounts of blood biomarkers, such as insulin growth factor (with normal to slightly high levels of IGF-1 generally associated with normal processes such as growth, metabolism, and cell proliferation) and interleukin 6 (with elevated levels of IL-6 generally associated with negative processes such as inflammation).

The paper describes IL6 and IGF-1 dynamics as a “*Hill-type kinetic law with the Hill coefficient (exponent for inhibitor concentration in the denominator) being 2. In chemical kinetics the Hill coefficient of more than 1 would signify positively cooperative binding—once an inhibitor binds, it enables easier binding of the next inhibitor*.” The paper also provides a differential equation for the production of IL6. But to a novice in modeling, neither this description nor the equation is easily comprehensible.

$$\frac{{\rm {d}}{{\rm {IL}}}6}{{{\rm {d}}t}}=\frac{{{kp}}_{{{\rm {IL}}}6}}{1+{({{ks}}_{{{\rm {IGF}}}1}{{\rm {IGF}}}1)}^{2}}-{{kd}}_{{{\rm {IL}}}6}{{\rm {IL}}}6$$

**Fig. 1:**
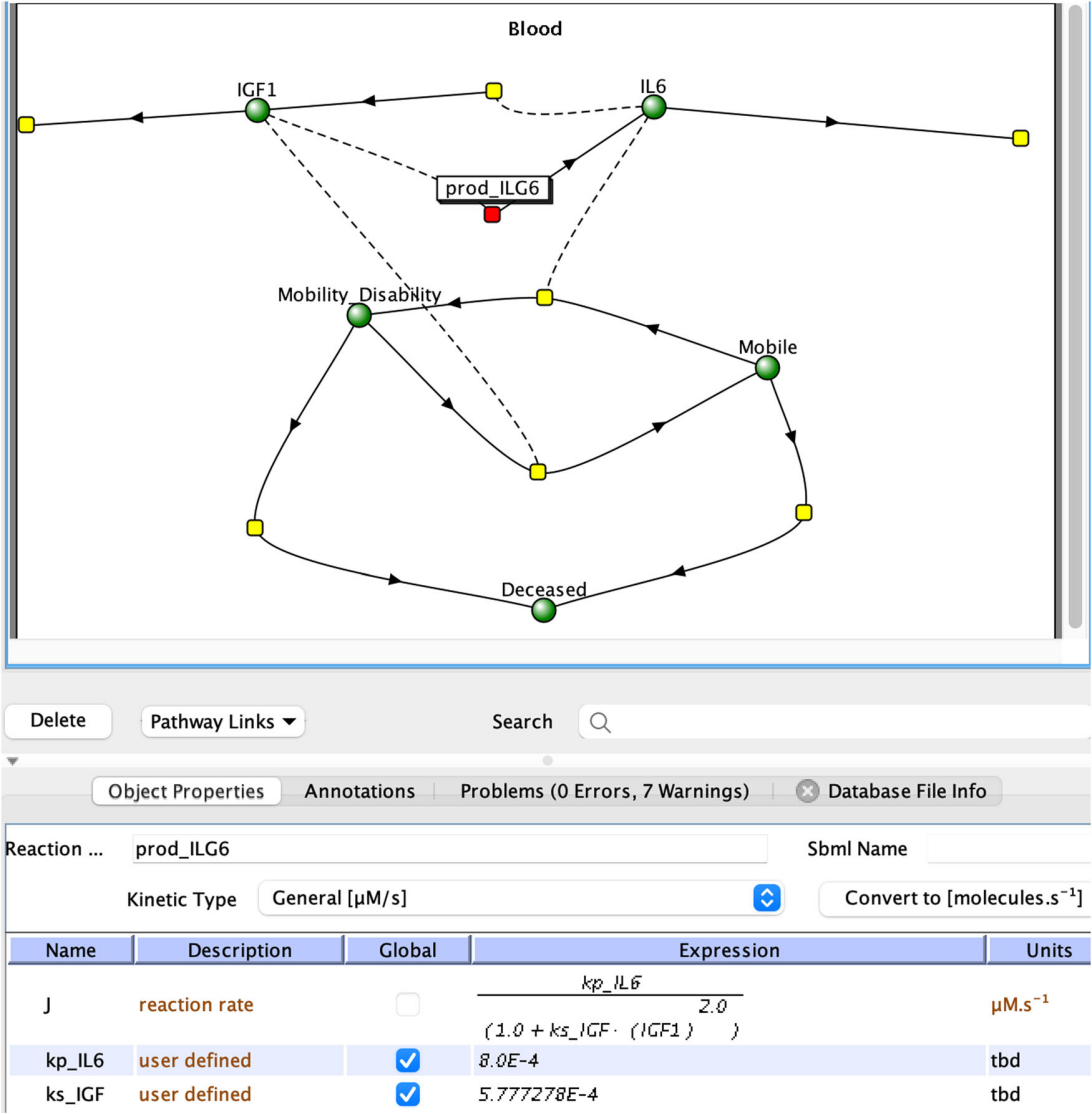
A visual representation of the Aging Phenotypes model in the Virtual Cell software (https://vcell.org^5,6^). The red node represents the reaction producing IL6. The bottom panel describes the expression for the reaction rate (called “J”).

To help understand the mathematical model, it can be loaded into the Virtual Cell (Vcell) software in the native VCML format provided in the manuscript (Supplementary Note 6). Figure 1 depicts the screenshot of model representation in the VCell interface, showing interactions among model elements such as IL6 and IGF1 and corresponding kinetic laws. However, a novice may need further help to interpret this cartoon, such as the node and line representation and the expression for reaction rate. Furthermore, no explanations were given in the paper or the associated VCML model as to the selection of the mathematical expressions, or of what they represent.

We then tested how AI can assist in explaining the model’s mathematics to a novice. The model in the VCML format, given in the paper’s supplemental material, was provided to ChatGPT (Supplementary Note 6), but the format was not recognized.

When the same model in the VCML format was provided to HyperWrite, the math in the model was described with great detail:

“*Based on the VCML model, the level of IGF1 affects the level of IL6 through the production of IL6 … IGF1 inhibits the production of IL6. Specifically:*

- When IGF1 levels are low (i.e., close to 0), the term (ks_IGF * (IGF1^2.0)) approaches 0, and the rate of IL6 production approaches its maximum value of kp_IL6.
- As IGF1 levels increase, the term (ks_IGF * (IGF1^2.0)) also increases, which decreases the rate of IL6 production.
- *At very high IGF1 levels, the rate of IL6 production approaches 0, indicating almost complete inhibition of IL6 production by IGF1*.

*Therefore, the model suggests that IGF1 negatively regulates IL6 production, meaning that higher levels of IGF1 lead to lower levels of IL6, and vice versa.”*

That is, the HyperWrite AI correctly analyzed the given mathematics, successfully identified the most important network information for the given model, and deduced an inhibitory relationship between IGF1 and IL6. Its summary statement drastically reduces the learning difficulties for a novice following the described aging study.

However, other AI tools provided incomplete or incorrect answers. MetaAI stated that *“IL6 production is induced by IGF1 and inhibited by IL6 itself, with a degradation term. IGF1 production is induced by IL6 and inhibited by IGF1.”* When asked a follow-up question to explain, “How does the level of IGF1 affect the level of IL6?” it gave the opposite answer “*IGF1 and IL6 have an inverse relationship, like two opposing forces. As one increases, the other decreases.”* Microsoft Copilot also incorrectly described the relationships between IGF-1 and IL-6 as “*The product is IGF1, influenced by the modifier IL6.” and “IL-6 enhances the production of IGF-1 by influencing its kinetics.”*

Taken together, at this stage, large language models implemented in ChatGPT, MetaAI, and Copilot are unsuitable for analyzing mathematical expressions in system biology. However, the HyperWrite AI may provide some success as exemplified above.

### Use of AI to explore model structures in system biology

Being able to accurately describe participants and their interactions in a biological system is fundamental to understanding a system biology model. Therefore, given a model description in the standard modeling formats, we tested whether AI tools can understand the structure of the mathematical model and whether AI can properly identify the composition and the reaction processes. In the test described below, we designed a series of simple mathematical models with increasing complexity.

**Fig. 2:**
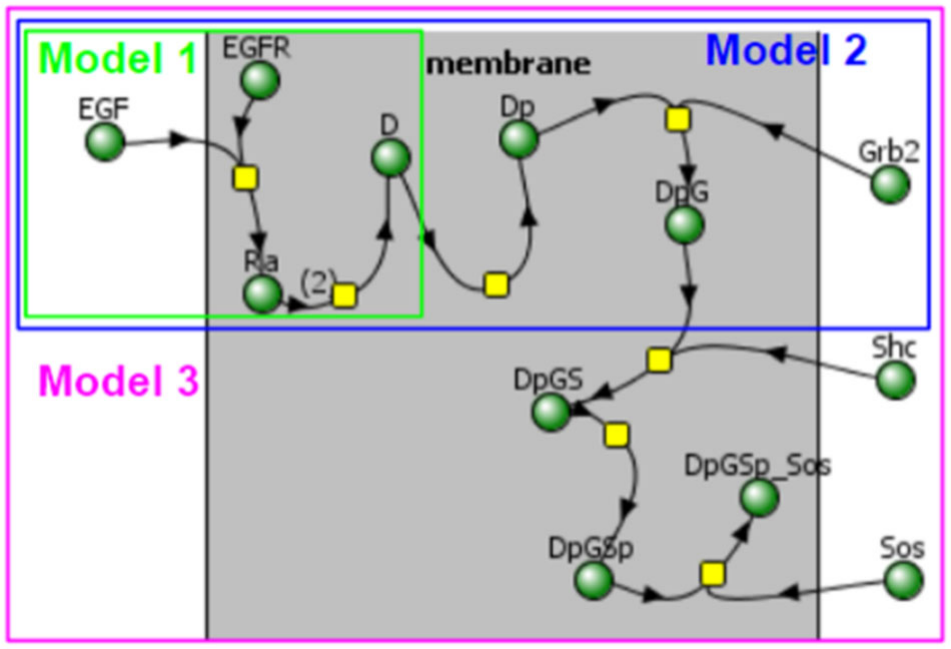
A typical representation of a mathematical model as a reaction diagram in the Virtual Cell software. Yellow nodes: reaction processes. Green nodes: chemical species participating in these processes. In Model 1, the EGF ligand’s binding to an EGFR receptor leads to the formation of a complex of EGF and EGFR in the membrane called Ra, and then the dimerization of two Ra complexes leading to the formation of a dimer D—a complex on a membrane consisting of two ligands EGF and two receptors EGFR. In Model 2, Model 1 is extended by adding a reaction of phosphorylation transforming D dimer into its phosphorylated form Dp, which binds with the Grb2 protein to form a DpG complex. In Model 3, Model 2 is extended by adding interactions with Shc and Sos proteins.

We started with the classical model of EGF ligand binding to the EGFR receptor, followed by its dimerization and phosphorylation (Fig. 2). This model has been used in multiple research studies^32–34^.

Next, we added levels of complexity with the aggregation of multiple proteins (namely Grb2, Shc, and finally SoS) on the membrane. As common in modeling, the names of introduced components were highly abbreviated: A species resulting from the binding of EGF and EGFR is named as Ra (stands for activated receptor), aggregation of two Ra species forming a species D (standing for dimer), a species resulting from phosphorylation of D called Dp (representing the phosphorylated state), aggregation of the Grb2 species with Dp resulted in the species named DpG, which would become DpGS when it is aggregated with the Shc protein, DpGSp when phosphorylated, and finally, DpGsp_Sos when the Sos protein is aggregated onto the compound.

Thus, the simplest Model 1 included only two reactions: binding of an EGF ligand to an EGFR receptor to form the activated complex Ra and Ra dimerization (Supplementary Notes 7, 8, vcml and sbml formats, respectively). Model 2 included 7 species, adding to the previous model the phosphorylation to Dp and aggregation with Grb2 to form DpG (Supplementary Notes 9, 10, vcml and sbml formats, respectively). Model 3 had 12 species, including Shc followed by Sos aggregating onto the protein complex (Supplementary Notes 11, 12, vcml and sbml formats, respectively).

These models were saved in VCML and SBML formats in the Virtual Cell software. Of note, the XML files are very verbose and free AI tools often limit the size and word count of input files. Thus, we created “simplified” versions of each of these model files in SBML format by deleting redundant parts of the code that did not directly relate to or hint towards the biological processes (e.g. units and parameters) (Supplementary notes 13–15).

With the designed mathematical models with increasing complexity, we tested whether AI can properly identify the composition of each species and the processes (binding, modification) that a species participates in. Table 2 summarizes the findings. Most tools, including ChatGPT, did not provide an understandable biology summary for the models, describing data structure such as unit definitions, parameters, and assignment rules. However, some tools were able to correctly describe the processes (reactions among species) and make sense of species names, such as understanding that Dp may be a phosphorylated form of D, and DpG may be a complex of Dp and G.

**Table 2:** A summary of AI description of model codes

AI tool	Model recognition features	M1	M2	M3
Meta AI	Recognized species names, e.g. that Dp might represent a phosphorylated form of the molecule D, DpG appears to be a compound that represents the complex formed by the interaction of Dp (a phosphorylated form of protein/molecule D) and Grb2.	P	P	P
Phind	Described interactions as conversions, e.g. describing aggregation of Dp and Grb2 as a “conversion of Dp into DpG, facilitated by Grb2”.	P	P	P
Perplexity	Correctly described small models, stating that Ra is a Receptor–ligand complex and dimerization of two activated receptor complexes forms a Dimerized receptor complex. However, for larger models Perplexity generated different answers and was never able to accurately describe what the model was depicting.	F	W	W

The columns M1, M2, M3 correspond to comprehension of Models 1–3 by AI, with F meaning Full, P—partial, and W—wrong. Other tools from Table 1 not listed here describe data structure only, providing no biological interpretation.

**Table 3:** A comparison of 10 AI platforms and their responses across seven common biological modeling formats

	Understanding of data provided in the following formats					
AI tool	SBML	BNGL	BioPAX	SBGN	VCML	NeuroML
ChatGPT	Mostly code	Correct	Correct	Correct	Missing details	Code only
Meta AI	Mostly code	Correct	Correct	Correct	Missing details	Correct
Gemini	Missing details	Not recognized	Not recognized	Correct	Correct	Correct
HuggingChat	Correct	Correct	Mostly code	Correct	File is too large	Code only
HyperWrite	Correct	Correct	Correct	Correct	Correct	Correct
Microsoft Copilot	Incorrect	Correct	Correct	Correct	Incorrect details	Correct
Phind	Missing details	Incorrect details	Code only	Correct	Missing details	Correct
Perplexity	Correct	Correct	Correct	Correct	Correct	Correct
SciSpace	Missing details	Incorrect	Code only	Correct	Incorrect	Missing details
You.com	Code only	Missing details	Not	Code only	File is too large	Code only

Based on tests as of July 2024. Provided is a short description of each platform’s performance for various formats. “Correct” is determined based on accurately identifying various species and reactions in the model, while “Missing details” represents a partial lack of these key species and reactions. “Mostly code” or “Code only” refer to answers solely focusing on the format and the code rather than providing a biological description. Answers that include incorrect details while also being partially correct are labeled “Incorrect details,” while completely incorrect answers are labeled “Incorrect.” If a platform cannot process or identify the format, it is labeled “Not recognized” while a lack of processing due to file length is labeled “File is too large.”

We conclude that some public AI tools can recognize and interpret key reactions and species in system biology models, but most AI tools struggle with biological interpretation and focus on the structure of the model and the code.

### Comparison of AI tools in handling various data formats

When using public AI tools in system biology, it is also crucial to compare AI’s capability to handle the diverse range of existing systems biology formats. By using the same workflow across multiple public AI platforms, we identified tools most suitable for specific tasks. Results are summarized in Table 3.

Overall, we found that the free public version of ChatGPT was the most effective among fully accessible AI platforms, while Perplexity gave better responses but had daily limits on use. Although Perplexity requires registration, its consistently high-quality answers across multiple systems biology formats, along with the fact that it provides references to the information it generates, were reasons why we ranked it as one of the best platforms. Both ChatGPT and Perplexity still had problems with at least one of the formats but for the most part, gave detailed and understandable answers.

HuggingChat was efficient in explaining models in SBML and BNGL formats while describing only the structure and few details in other formats. All the answers provided by You.com concentrated on file structures only and did not explain the biological details of the models.

Gemini, Meta, HyperWrite, and Phind were able to provide high-quality descriptions for some systems biology formats and responded with brief or partially false answers with others. Meta, for example, identified key interactions in BioPAX such as *“Ligand binding: EGF-like ligands bind to EGFR”* and *“Receptor dimerization: Two EGFR molecules bind to form a dimer.”* Gemini generated good responses for SBGN, VCML, and NeuroML identifying all the key components in the models and providing detailed and concise answers in a bulleted format. It explained SBML models, *“The code you provided describes a biochemical pathway involving a molecule called EGF (Epidermal Growth Factor). EGF binds to a receptor (EGFR) on the surface of a cell. This binding can trigger a series of reactions inside the cell”*. HyperWrite, similarly to MetaAI, did a good job with BioPAX and was able to discuss EGFR dimerization as “*two instances of a protein complex composed of EGFR and EGF-like ligands, which are bound to the plasma membrane…combine to form the product.”* Phind was relatively strong with SBML, describing the model and highlighting “*the process of EGFR (Epidermal Growth Factor Receptor) dimerization*” but not describing the specific species involved in the reaction. HyperWrite was the only AI tool in our tests that could properly understand and explain Math expressions in XML formats. Microsoft Copilot provided a good analysis of many formats, but could also give completely irrelevant explanations, e.g. describing the aging model from Supplementary Note 6 as *“Fluid Mosaic Model”*. And SciSpace’s answers were generic and concentrated on the data structure rather than on the biological content, but it was able to recognize multiple formats and generate meaningful brief responses. However, for formats not recognized, SciSpace provided answers and sources that were completely irrelevant.

Based on our tests, the SBML format was the most compatible across different AI platforms, which is not surprising given the wide acceptance of models in this format. Every AI application was able to process SBML, but the quality differed based on the size and levels of complexities of user input files.

### Proposed approach/workflow for using AI tools in systems biology

**Fig. 3:**
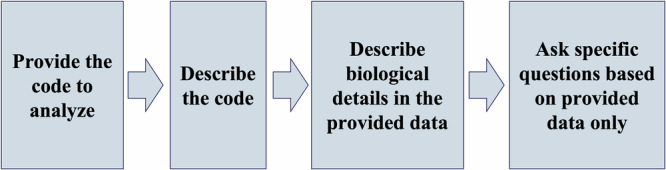
A proposed workflow for questioning AI. The workflow is composed of a series of questions that were used when analyzing the Aging Phenotypes model in VCML format.

AI is evolving and learning all the time. As a result, the following proposed workflow (Fig. 3) may be outdated later on, but it still can serve as a guideline for questioning AI to obtain desired results.

Please note that asking questions to ChatGPT (or any AI-based conversational model) is not as straightforward as it may seem. When asking questions to AI the user must be as specific and clear as possible, provide relevant context or background, break down complex queries into smaller parts, and iterate and refine questions if the first response doesn’t meet your needs.

AI remembers previous questions during the session, so the user may need to restart or use a new session in an incognito browser to prevent bias from the previous questions. One of the significant issues working with AI tools is that they are trained on a large body of knowledge that may be contradicting to the data contained in the systems biology resource (model, pathway, diagram). To overcome this issue, there are several strategies:

1. Specify a data input that has to be used and ask to use only it, e.g., “Using only the information provided below, summarize it in biological terms”
2. If needed, ask to condense the answer: “Summarize the following code in less than 100 words, focusing on chemical species and interactions among them, and exclude technical details about the format used”.
3. If needed, ask to skip details about the data format.
4. Ask specific questions like “what are interactions in the model”, “what is the rate of change for a give species”, always mentioning “use the data above”, and reminding about conciseness when needed.

For example, to inquire about the details of the biological mechanism in the model coded in SBML format, we had to enter the sequence of questions:

Question 1: Describe chemical species and interactions among them for the model described in: [input data].

Question 2: Describe biological pathways related to [input data] only.

Question 3: Based only on the information above, describe relationships between [species A] and [species B].

Question 4: Based only on the information above, describe all interactions of [species A] in the model.

Question 5: Based only on the information above, explain the meaning of the reaction mathematics for the [interaction name].

Question 6: Based only on the information above, explain how is [species A] related to [species B]

Sometimes, users need to enforce questions several times, requiring the AI to concentrate: *“Please briefly describe only biological mechanisms in the file above, skipping all details related to simulations.”*

The responses by AI need to be closely monitored and compared, as they are highly dependent on the small variations in the questions asked. Anecdotally, there may be very drastic differences in AI responses to “Describe interactions in [input data]” versus “Describe interactions in the following text [input data]”.

Note that different AI tools could use different questions or different numbers of questions to achieve a similar concise description of biology in the supplied data. Some tools like HyperWrite, SciSpace, and You.com provided a reasonable description of biological mechanisms after one question. However, many AIs discussed the format structure and needed additional questions to describe the biological processes. Question 4 proved especially useful in refocusing AIs, allowing them to provide detailed answers focusing solely on the species and reactions in a model rather than any redundant information about the code itself. Some AI tools never resolved to the textual description and always provided answers concentrating on a data structure rather than providing a biological description.

## Discussion

The overall goal of this paper was not to provide a comprehensive comparative analysis of various AI tools but rather to provide an overview of the capabilities of public AI tools as a whole through a systems biology lens. We concentrate on the analysis of systems biology resources in mathematical modeling, and specifically on using public AI to analyze data stored in the formats supported by the “COmputational Modeling in BIology NEtwork” (COMBINE) initiative to coordinate the development of the various community standards for computational models. We found that, overall, public AI could hold many benefits if used for novice exploration of the field. By testing systems biology data in multiple formats vs. AI tools, we found that in most cases public AI was successfully capable of identifying the most important information and presenting it in a human-readable format, which is valuable in dealing with complex models. However, each AI has different pros and cons. Platforms such as Meta AI can identify key species in modeling files from abbreviated names, such as Dp representing a phosphorylated D molecule in the EGFR model. HyperWrite was able to correctly analyze the mathematics in the files describing the mathematical models and properly explain reasons for selecting specific mathematical expressions. These are conclusions that a novice in system biology may have difficulties drawing independently when provided with just the data. Being able to comprehend complex biological and mathematical equations and present them in a human-readable format could prove to be very useful for a novice looking to learn and explore systems biology models. Based on our tests, the formats that were most commonly used in various studies (such as SBML) were most understandable by various public AI tools.

On the other hand, it is important to consider that using public AI may lead to incorrect conclusions. Some tools correctly identified biological sequences, while other tools claimed different ones. Thus, it is important to always take AI-generated responses with some hesitancy. Moreover, even within a single AI platform, there is no reproducibility. The same question asked in a slightly different way may generate completely different answers. Even further, asking the same question again can generate a completely new response. Vigilance is important when using public AI to catch these errors, and repeatedly questioning AI to generate different responses can be helpful when searching for a desired outcome.

File size and truncation issues have also to be noted in our tests. When using the free versions of various public AI platforms, there are often limits on file size, even if not explicitly shown to the user. Perplexity demonstrated how larger file sizes can cause problematic issues for AI systems, by generating inaccurate and inconsistent answers at larger complexity levels in the EGFR model. The answers varied from those produced with the smaller files, as well as those produced with the other variations of the larger file size. Another observation for public AI tools is that although they generally provided accurate and detailed responses, they often included some false or superficial details in their responses and could be inconsistent when repeatedly questioned.

When using AI for analysis, it is crucial to compare how different platforms can handle the same data. By using the same workflow across multiple public AI platforms, a user has a higher chance of preventing errors and false information in their answers.

While using public AI for understanding systems biology resources is feasible and useful, using public AI for other potential applications, such as expanding mathematical models in biology, or performing novel analysis, is still not recommended. Tasks such as “Create an SBML model of interactions among ligand and receptor” can be performed by most public AI (and ChatGPT specifically) correctly. The AI-generated models are useful for learning modeling and modeling formats. De-novo modeling, expanding mathematical models, or model analysis require user-defined assumptions and data, specific to these assumptions. Precise formulation of assumptions is often as difficult as the design of a model itself.

Another issue with using public AI for understanding and designing systems biology models is the insufficient amount of systems biology data in the standards discussed in the manuscript. There are several thousand models in modeling databases, which may be insufficient for the proper AI training. Thus, mathematical models generated by AI will have errors. However, Microsoft CoPilot, designed to assist in coding, serves as an example of AI potential for mathematical modeling. AI can work in a more supportive role, providing clues about new elements that can be added to the models or suggesting explanations for the observed simulation results.

To conclude, public AI tools demonstrate promise in explaining to users the various types of systems biology data, while also significantly reducing the burden of learning different data formats, database interfaces, and software tools. If users pay attention to any inconsistencies and cross-check the responses, freely accessible AI tools can be valuable learning devices that can assist non-data science users looking to explore systems biology data.

## Methods

Input files used in our tests and analyses are described here and also available in the supplemental material.

A VCML file (Supplementary Note 1) was obtained from the supplemental material of the paper by Kaste et al. ^35^ and relevant parts of the VCML code were input into AI tools to check if not well-documented nonhuman-readable formats could be converted into understandable summary, including key biological information. The BioPAX and SBGN files of the EGFR pathway (Supplementary Notes 2 and 4, respectively) were downloaded from the Reactome database (https://reactome.org/content/detail/R-HSA-177922). BioPAX is a very verbose format (61 kB compared to 2 kB for SBGN description of the same process), so some tools could not accept it, truncated, or accepted piecewise. The NeuroML file (Supplementary Note 3) describing a simple cell with a morphology was downloaded from the NeuroML website (https://docs.neuroml.org/Userdocs/NeuroMLv2.html). This is a concise file that has no annotations or descriptions of processes. The BNGL file of the EGFR pathway (Supplementary Note 5) was a tutorial example downloaded from the bnglViz website (https://bnglviz.github.io/examples.html). The VCML file of the Aging Phenotypes model (Supplementary Note 6) was saved from the VCell list of published models (https://vcell.org/biomodel-245335415). The generated Models 1–3 describing the EGFR signaling pathway with various levels of complexity and reactions were created in the Virtual Cell software and exported in both VCML and SBML formats. Model 1 (Supplementary Notes 7 for VCML and 8 for SBML) had its SBML code “simplified” and condensed to create a smaller file for easier AI processing (Supplementary Note 13). Model 2 (Supplementary Notes 9 for VCML and 10 for SBML) and Model 3 (Supplementary Notes 11 for VCML and 12 for SBML) each had their own “simplified” SBML files created as well (Supplementary Notes 14 and 15, respectively).

## Supplementary information

Supplemental material

## Data Availability

No datasets were generated or analyzed during the current study.
