# Supplementary material for: Leveraging public AI tools to explore systems biology resources in mathematical modeling
Source: NPJ Syst Biol Appl. 2025 Feb 4;11:15. doi: 10.1038/s41540-025-00496-z (PMC11799200; doi:10.1038/s41540-025-00496-z)
Supplement: Supplementary file 1 — Supplemental material [file 41540_2025_496_MOESM1_ESM.zip › Supplemental Material/Supplemental material.docx]

Supplemental material

Suppl 1: VCML: <https://github.com/mjohn218/dynamin_model/blob/main/VirtualCellInputs/DiffuseToCluster_BB_PostStim.vcml>

Suppl. 2: BioPAX: <https://reactome.org/ReactomeRESTfulAPI/RESTfulWS/biopaxExporter/Level2/177922>

Suppl. 3: NeuroML: <https://github.com/NeuroML/NeuroML2/tree/master/examples/NML2_SimpleMorphology.nml>

Suppl. 4: SBGN: <https://reactome.org/ContentService/exporter/event/R-HSA-177922.sbgn>

Suppl. 5: BNGL: <https://bnglviz.github.io/models/Blinov_egfr.bngl>

Suppl. 6: VCML: <https://vcell.cam.uchc.edu/api/v0/biomodel/245335415/biomodel.vcml>

Suppl. 7: <https://vcell.org/bionetgen/SupplMat/Suppl7_model1.vcml>

Suppl. 8: <https://vcell.org/bionetgen/SupplMat/Suppl8_model1.xml>

Suppl. 9: <https://vcell.org/bionetgen/SupplMat/Suppl9_model2.vcml>

Suppl. 10: <https://vcell.org/bionetgen/SupplMat/Suppl10_model2.xml>

Suppl. 11: <https://vcell.org/bionetgen/SupplMat/Suppl11_model3.vcml>

Suppl. 12: <https://vcell.org/bionetgen/SupplMat/Suppl12_model3.xml>

Suppl. 13: <https://vcell.org/bionetgen/SupplMat/suppl13_model1_simplified.xml>

Suppl. 14: <https://vcell.org/bionetgen/SupplMat/Suppl14_model2_simplified.txt>

Suppl. 15: <https://vcell.org/bionetgen/SupplMat/suppl15_model3_simplified.txt>
